# Bilateral Primary Angiosarcoma of the Breast

**DOI:** 10.1155/2013/139276

**Published:** 2013-09-30

**Authors:** P. Keshav, Shruti S. Hegde

**Affiliations:** ^1^Associate Professor, Department of Surgery, KMC, Mangalore, Manipal University, Karnataka 575003, India; ^2^KMC, Mangalore, Manipal University, Karnataka 575003, India

## Abstract

Primary breast sarcomas are very rare entities, accounting for 0.04% of all malignant neoplasms. Angiosarcoma of breast is infrequent and is an endothelial malignant tumor with bad prognosis because of the frequency of metastasis and recurrence. We present a case of a 30-year-old female who presented with an ulcerated left breast lesion which on further workup revealed to be a primary angiosarcoma of breast with metastasis to right breast.

## 1. Introduction

Angiosarcomas are uncommon malignant neoplasms characterized by rapidly proliferating and extensively infiltrating anaplastic cells derived from blood vessels and lining irregular, blood-filled spaces. The term angiosarcoma is applied to a wide range of malignant endothelial vascular neoplasm's that affect a variety of sites. Angiosarcomas are aggressive and tend to recur locally and spread widely and have a high rate of lymph node and systemic metastases.

## 2. Case Report

**Figure 1:**
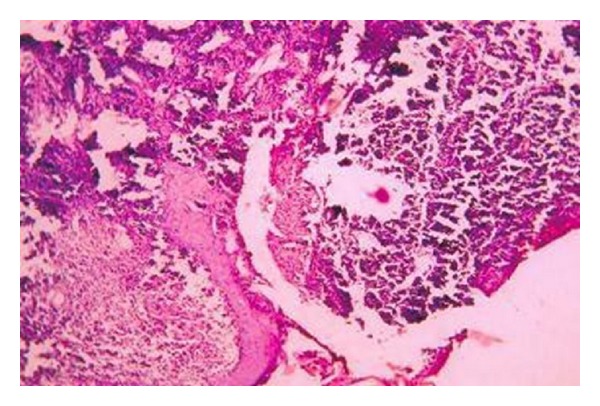
Histological appearance under low magnification.

**Figure 2:**
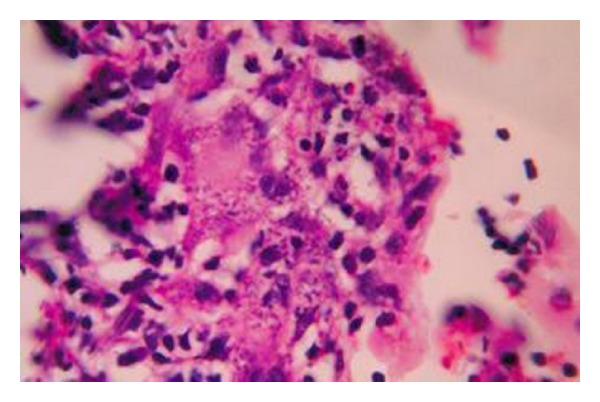
Histological appearance under high magnification.

A 30-year-old unmarried lady presented with an ulcerated lump in the left breast. Initially, patient had noticed a peanut sized painless swelling in her left breast six months back which rapidly increased in size and the skin over the swelling spontaneously ulcerated with seropurulent discharge with occasional bleeding from the ulcer. There was no history of breast surgery or breast irradiation. On examination the lump was 8 cms × 8 cms in diameter and was fixed to skin but free from underlying muscle and chest wall. There were multiple enlarged axillary lymph nodes with the largest measuring 3 cms × 3 cms. In the opposite breast, a lump measuring 5 cms in diameter was detected which the patient had failed to notice. Axillary nodes were palpable on the right side as well. A provisional diagnosis of stromal sarcoma of the breast was considered after a Fine Needle Aspiration Cytology (FNAC) from both lumps. The patient underwent bilateral modified radical mastectomy with axillary clearance. The histology revealed multiple irregular vascular spaces of different sizes lined by single layer of endothelial cells which were surrounded by groups, bundles and masses of oval, and spindle shaped and pleomorphic cells having ovoid and pleomorphic hyperchromatic nuclei (Figures 1 and 2). The histological feature was consistent with the bilateral Angiosarcomas of breast. Lymph nodes, however, showed reactive hyperplasia.

## 3. Discussion

Angiosarcoma of the breast is a rare and highly lethal neoplasm accounting for less than 0.1% of malignant breast tumors [1]. All Angiosarcomas tend to be aggressive and often are multicentric. Malignant vascular tumors are clinically aggressive and difficult to treat and have a reported 5-year survival rate of less than 20% and a median survival of just 22 months [2]. Advanced stage at presentation and lack of extensive excision are associated with higher recurrence, distant metastasis rates, and worsened survival. This malignant tumor occurs primarily in young women, with 6% to 12% of the cases found during pregnancy, implying a hormonal effect [2]. Preoperative diagnosis of angiosarcoma of the breast by aspiration cytology and biopsy is often difficult, with a false-negative biopsy rate of 37% in one large review [3].

In most cases, the tumor is larger than 4 cm in diameter. In a series at Mayo Clinic tumor size was a more valuable prognostic factor than tumor grade [4]. These rapidly growing lesions often arise deep within breast tissue, causing diffuse breast enlargement with associated bluish skin discoloration. They usually spread locally as ill-defined, hemorrhagic, spongy masses. Unlike breast carcinomas, skin retraction, nipple discharge, and axillary lymphnode involvement are absent. In our case, however, we noticed that the skin was involved, but the lymph nodes were negative for malignancy. An exceptional case old male breast angiosarcoma has also been described [5].

Low grade angiosarcomas are readily mistaken for benign hemangioma. Angiolipoma features intermingling vessels and fat lobules which may be mistaken for invasion of fat by an angiosarcoma. Papillary endothelial hyperplasia as described by Branton et al. is the “great imposter for angiosarcoma” [6]. The treatment of angiosarcoma of the breast is early and complete surgical excision of the mass with tumor-free margins because of the neoplasm often extends microscopically beyond its gross limits. Adjuvant chemotherapy that includes doxorubicin for patients with poorly differentiated angiosarcoma of the breast results in a higher proportion of patients who are relapse-free compared to patients not receiving adjuvant chemotherapy [7]. Radiotherapy is reserved after lumpectomy, and following total mastectomy if the tumor is larger than 5 cm, the margins are positive, or if the skin or regional nodes are affected.
